# α_1B_-Adrenergic receptor signaling controls circadian expression of *Tnfrsf11b* by regulating clock genes in osteoblasts

**DOI:** 10.1242/bio.012617

**Published:** 2015-10-09

**Authors:** Takao Hirai, Kenjiro Tanaka, Akifumi Togari

**Affiliations:** Department of Pharmacology, School of Dentistry, Aichi-Gakuin University, Nagoya 464-8650, Japan

**Keywords:** α_1B_-adrenergic receptor, OPG, Bmal1, REV-ERBα, Osteoblast

## Abstract

Circadian clocks are endogenous and biological oscillations that occur with a period of <24 h. In mammals, the central circadian pacemaker is localized in the suprachiasmatic nucleus (SCN) and is linked to peripheral tissues through neural and hormonal signals. In the present study, we investigated the physiological function of the molecular clock on bone remodeling. The results of loss-of-function and gain-of-function experiments both indicated that the rhythmic expression of *Tnfrsf11b*, which encodes osteoprotegerin (OPG), was regulated by Bmal1 in MC3T3-E1 cells. We also showed that REV-ERBα negatively regulated *Tnfrsf11b* as well as *Bmal1* in MC3T3-E1 cells. We systematically investigated the relationship between the sympathetic nervous system and the circadian clock in osteoblasts. The administration of phenylephrine, a nonspecific α1-adrenergic receptor (AR) agonist, stimulated the expression of *Tnfrsf11b*, whereas the genetic ablation of α_1B_-AR signaling led to the alteration of *Tnfrsf11b* expression concomitant with *Bmal1* and *Per2* in bone. Thus, this study demonstrated that the circadian regulation of *Tnfrsf11b* was regulated by the clock genes encoding REV-ERBα (*Nr1d1*) and Bmal1 (*Bmal1*, also known as *Arntl*), which are components of the core loop of the circadian clock in osteoblasts.

## Abstract (precis)

**Summary:** This study demonstrates that the circadian regulation of *TNFRSF11B* is regulated by the clock genes *Nr1d1* and *Arntl*, which are components of the core loop of the circadian clock in osteoblasts, providing a molecular mechanism for the control of bone remodelling by circadian rhythms.

## INTRODUCTION

Circadian clocks are endogenous and biological oscillations that occur with a period of <24 h and coordinate behavioral and biochemical processes with the day/night cycle. The circadian clock in each of these tissues plays a critical role in the daily patterning of diverse physiological processes such as sleep/wake cycles, feeding, and metabolism (Dibner et al., 2010; Mohawk et al., 2012; Yamaguchi et al., 2013). In mammals, the central circadian pacemaker is localized in the suprachiasmatic nucleus (SCN) of the hypothalamus and synchronized primarily through phonic signals, whereas peripheral oscillators may be adjusted by neural and hormonal signals (Mohawk et al., 2012). Peripheral tissues, including bone and cartilage, are also autonomous circadian oscillators (Yoo et al., 2004; Dibner et al., 2010; Okubo et al., 2013) and have centrally coordinated functions. On the other hand, previous studies identified a link between the SCN and peripheral tissues through the sympathetic nervous system (Buijs et al., 1999; Terazono et al., 2003). Recent studies indicated that SCN-controlled circadian hormonal rhythms and sympathetic tone played a central role in biological oscillations in bone (Fujihara et al., 2014; Swanson et al., 2015).

A molecular clock in the SCN and peripheral tissues has been shown to control the daily rhythms of physiology, metabolism, and behavior (Bass and Takahashi, 2010). This molecular clock is composed of a set of multiple clock genes, such as circadian locomotor output cycles protein kaput (CLOCK), brain and muscle Arnt-like protein-1 (Bmal1), PERIOD (PER), CRYPTOCHROME (CRY), REV-ERBα, and retinoic acid-related orphan receptor α (RORα) and their proteins, which are required for the generation of endogenous circadian oscillations (Ko and Takahashi, 2006). At the cellular level, autonomous rhythms are defined as a transcriptional and translational feedback loop oscillator involving cis-regulatory elements such as E-boxes, D-boxes, and ROR-elements (RORE) (Ueda et al., 2005; Mohawk et al., 2012). The core loop consists of the transcriptional activator the Bmal1 and CLOCK heterodimer, which activates the transcription of PER and CRY. PER and CRY proteins form heterodimeric complexes that inhibit their own transcription by suppressing the activity of Bmal1–CLOCK (Mohawk et al., 2012). REV-ERBα and REV-ERBβ are nuclear receptors that stabilize the loop within the clockwork by Bmal1–CLOCK transactivation of the nuclear receptors RORα and REV-ERBα, which feedback to activate or repress Bmal1 transcription through competition for shared RORE promoter elements and regulate the expression of genes involved in the control of circadian rhythms and metabolism (Marciano et al., 2014). Clock-controlled genes (CCGs) also modulate essential physiological processes: cell division (Fu and Lee, 2003), the control of metabolism by nuclear receptors (Yang et al., 2006), and the modulation of T_H_17 cell differentiation (Yu et al., 2013). Furthermore, previous studies indicated that the circadian clock was intimately associated with pathophysiological responses to environmental stress (Fu and Lee, 2003; Zheng et al., 2007; Keller et al., 2009; Sukumaran et al., 2011).

Bone is a metabolically active tissue that undergoes repeated cycles of bone remodeling including osteoblast-mediated bone formation and osteoclast-mediated resorption. Osteoprotegerin (OPG), a secreted glycoprotein belonging to the TNF superfamily, inhibits the formation, function, and survival of osteoclasts by preventing the binding of the RANK ligand (RANKL) to RANK (receptor activator of NF-kB), primarily as a soluble decoy receptor (Simonet et al., 1997). Several lines of evidence have demonstrated that the RANKL–RANK–OPG pathway plays critical roles in bone homeostasis through the regulation of osteoclasts (Lacey et al., 1998; Yasuda et al., 1998). Recent studies showed the biological functions of the RANKL–RANK–OPG system in development, immunity, and disease and its potential as a target for therapeutic agents in the treatment of osteoporosis and cancer (Baud'huin et al., 2013; Danks and Takayanagi, 2013; Walsh and Choi, 2014; Sigl and Penninger, 2014). However, the relationship between the molecular clock and RANKL–RANK–OPG system in bone remodeling remains unclear. In the present study, we demonstrated that the circadian regulation of *Tnfrsf11b*, which encodes OPG, was achieved by the clock genes REV-ERBα and Bmal1, which are components of the core loop of the circadian clock in osteoblasts. We also systematically investigated the relationship between the circadian clock and α_1B_-adrenergic receptor (AR) signaling in osteoblasts. These results provide a molecular mechanism for the control of bone remodeling by circadian rhythms.

## RESULTS

### Circadian expression of *Nr1d1* in bone

**Fig. 1.**
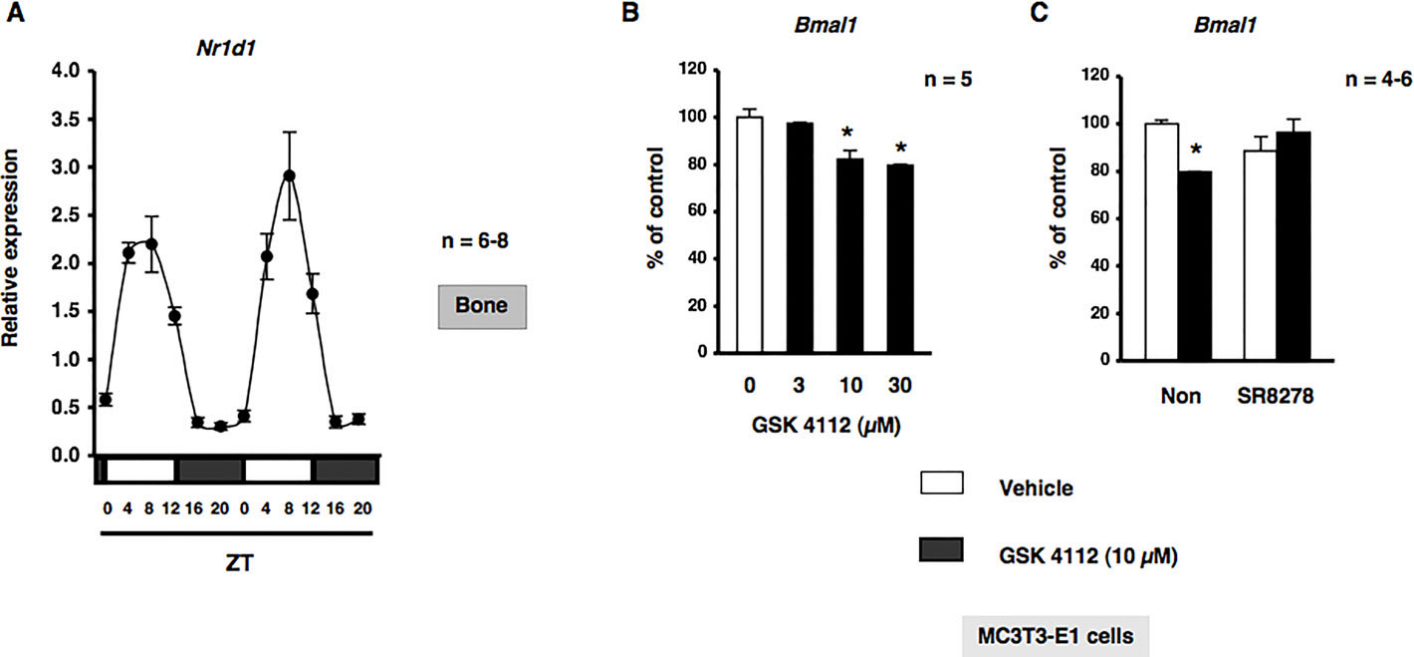
**REV-ERBα negatively regulates Bmal1 expression in MC3T3-E1 cells.** (A) A representation of the expression of *Nr1d1* in femurs (cancellous and cortical bone) from C57BL/6J mice under light/dark cycle conditions. Bone was obtained from C57BL/6J mice every 4 h. Total RNA was isolated, and the level of mRNA was determined by real time qRT-PCR using specific primers for *Nr1d1*. Relative mRNA levels were normalized to *Gapdh* levels. Data represent the mean±s.e.m, *n*=6-8 animals. A representative result of three individual experiments is shown. White boxes, light period; black boxes, dark period. (B) REV-ERBα negatively regulates *Bmal1* expression in MC3T3-E1 cells. *Bmal1* mRNA was down-regulated by GSK4112 in a concentration-dependent manner in MC3T3-E1 cells. Cells were treated with GSK4112 at 3 to 30 µM for 12 h, harvested and processed for real time qRT-PCR. Each value represents the mean±s.e.m. of five separate experiments. *P<0.05, significantly different from each control value obtained in MC3T3-E1 cells cultured in the presence of DMSO (vehicle control). (C) Cells were incubated for 12 h in the presence of GSK4112 with SR8278 at a concentration of 10 µM, followed by the determination of *Bmal1* levels by real time qRT-PCR. Each value represents the mean±s.e.m. of three or four separate experiments. *P<0.05, significantly different from each control value. NS, not significant.

In order to obtain a deeper understanding of the physiological function of the circadian clockwork on bone metabolism, we initially evaluated the expression of canonical core clock genes in bone. We previously reported that the expression of *Bmal1* and *Per2* displayed a circadian pattern over 24 h with opposing phases in bone (Hirai et al., 2014b). Therefore, we examined the expression of *Nr1d1*, which encodes REV-ERBα, in cancellous and cortical bone. As shown in Fig. 1A, the rhythmic expression of *Nr1d1* peaked near zeitgeber time (ZT) 8 in a 24-h rhythm in mouse bone samples harvested during a circadian cycle. To further understand the physiological function of the circadian clock and detect the circadian core system controlled by REV-ERBα in osteoblasts, we evaluated the expression of *Bmal1* with or without treatments with GSK4112 and SR8278, which are a synthetic REV-ERB agonist and antagonist, respectively (Grant et al., 2010; Kojetin et al., 2011). Total RNA was extracted from MC3T3-E1 osteoblastic cells following exposure to GSK4112 for 12 h, and was subsequently analyzed by real time qRT-PCR. As shown in Fig. 1B, the expression of *Bmal1* mRNA significantly decreased in a concentration-dependent manner in MC3T3-E1 osteoblastic cells. In addition, the pretreatment with the REV-ERB antagonist SR8278 completely inhibited the GSK4112-induced expression of *Bmal1*, as determined by a real time qRT-PCR analysis in MC3T3-E1 osteoblastic cells, which suggested that *Bmal1* was negatively regulated by REV-ERBα in osteoblasts (Fig. 1C).

### *Tnfrsf11b* was regulated by the circadian core system in MC3T3-E1 osteoblastic cells

**Fig. 2.**
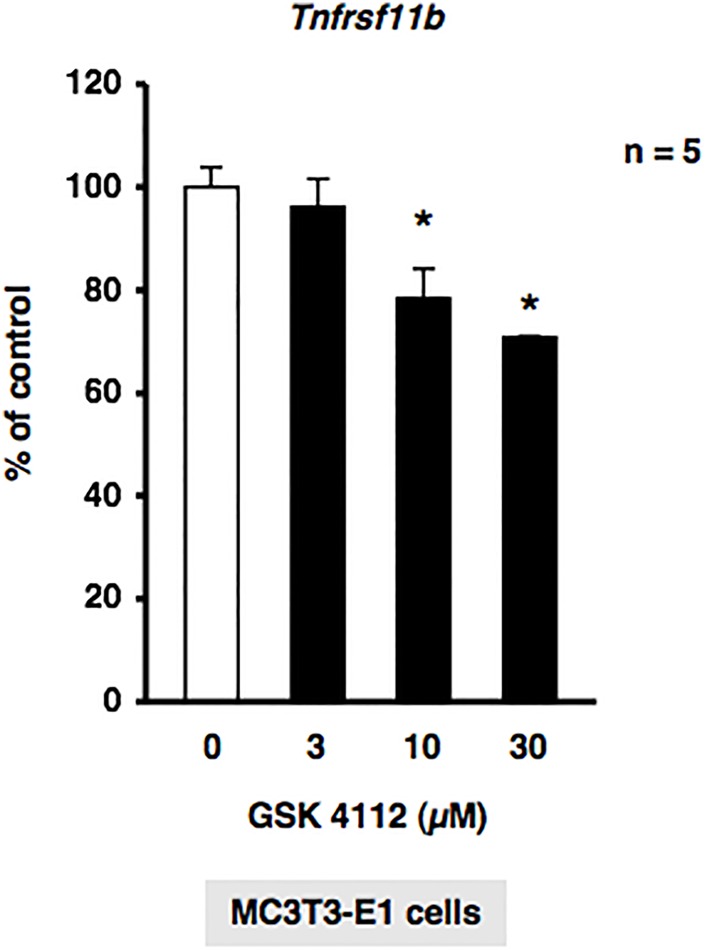
**GSK4112 suppressed *Tnfrsf11b* gene expression in MC3T3-E1 cells.** *Tnfrsf11b* mRNA was down-regulated by GSK4112 in a concentration-dependent manner in MC3T3-E1 cells. Cells were treated with GSK4112 at 3 to 30 µM for 12 h, harvested, and processed for real time qRT-PCR. Each value represents the mean±s.e.m. of five separate experiments. *P<0.05, significantly different from each control value obtained in MC3T3-E1 cells cultured in the absence of GSK4112.

**Fig. 3.**
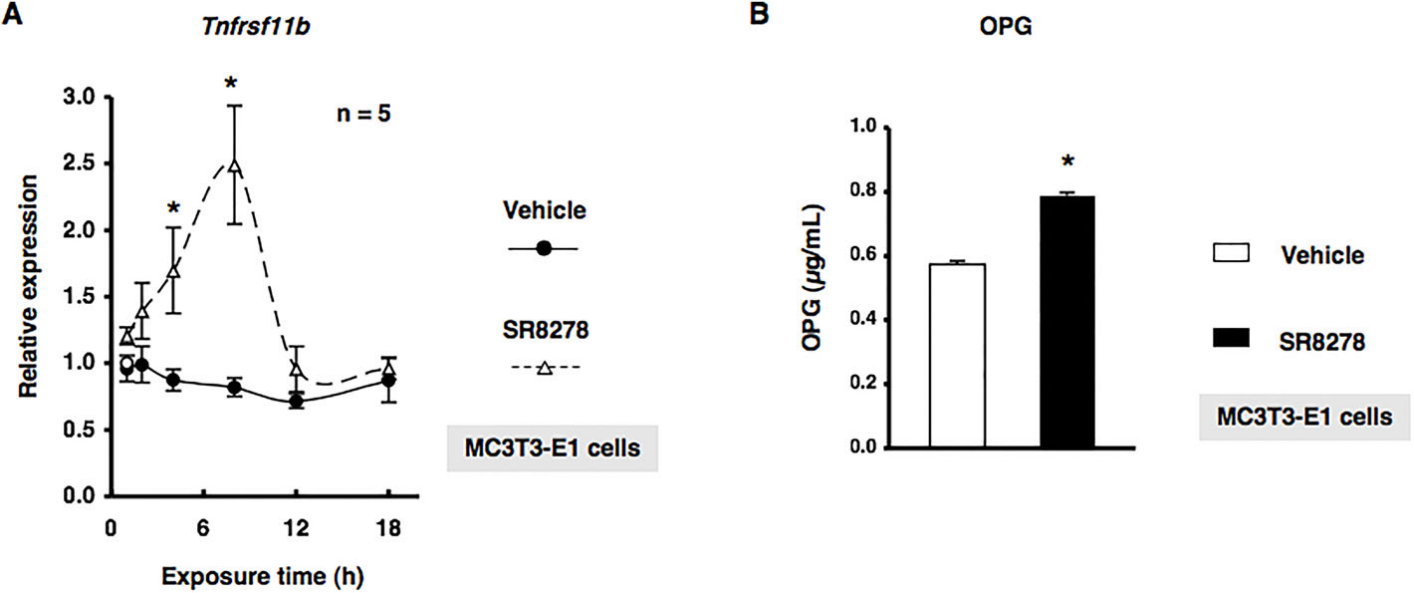
**The REV-ERB antagonist SR8278 up-regulated OPG in MC3T3-E1 cells.** (A) Cells were treated with 10 µM SR8278 or DMSO (Vehicle) for 1, 2, 4, 8, 12, and 18 h, harvested, and then processed for real time qRT-PCR. Each value represents the mean±s.e.m. of five separate experiments. *P<0.05, significantly different from each control value obtained in MC3T3-E1 cells cultured in the presence of DMSO. A representative result of three individual experiments is shown. (B) Cells were treated with 10 µM SR8278 or DMSO (Vehicle) for 24 h, and media was then collected for the OPG ELISA assay. Each value represents the mean±s.e.m. of four separate experiments. *P<0.05, significantly different from control value obtained in the absence of SR8278.

**Fig. 4.**
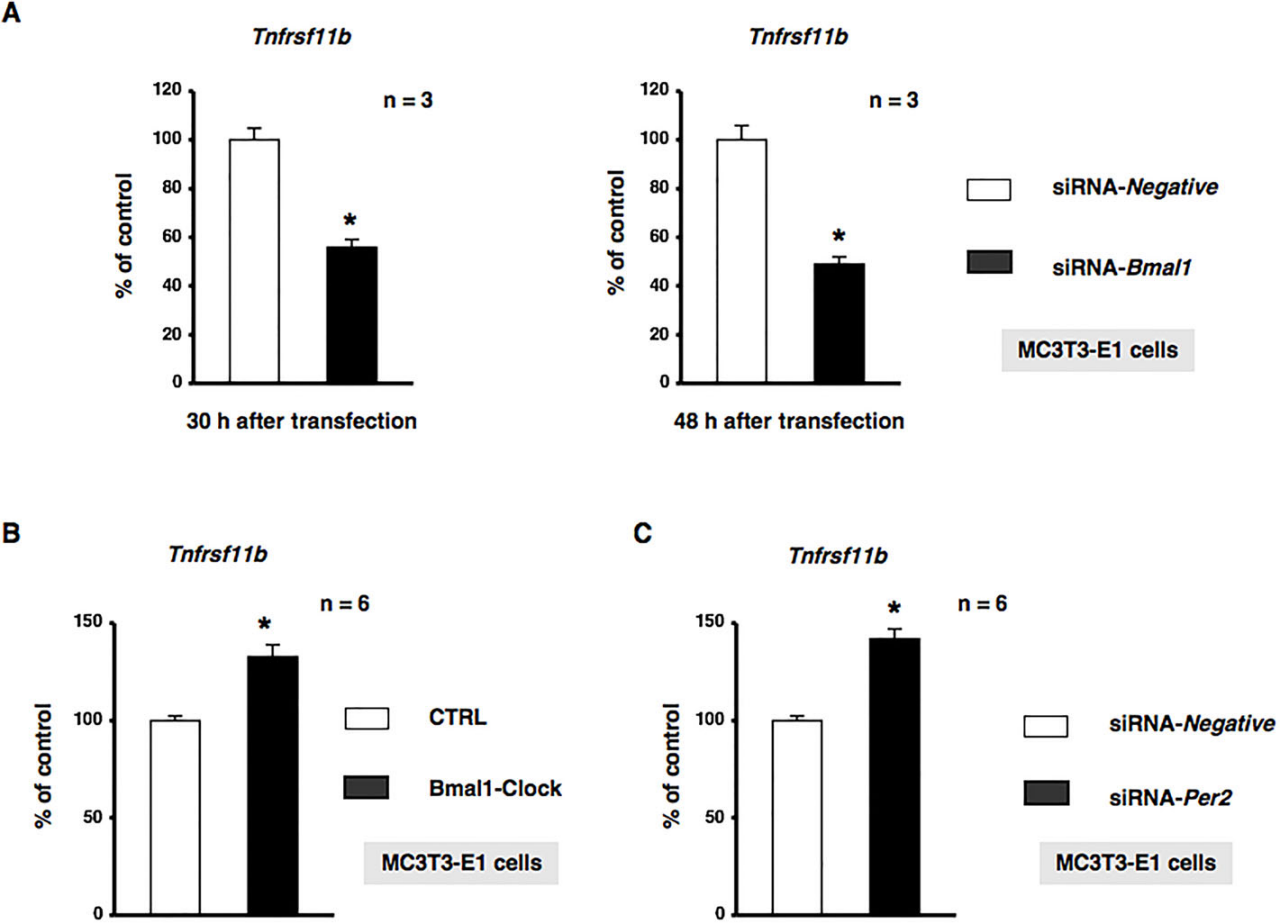
***Tnfrsf11b* was regulated by core clock genes in MC3T3-E1 cells.** (A) Bmal1-knockdown by siRNA in MC3T3-E1 cells. MC3T3-E1 cells were treated with Bmal1 siRNA (siRNA-*Bmal1*) or non-silencing RNA (siRNA-*Negative*), followed by further cultivation for 30 h (left panel) and 48 h (right panel) and the subsequent determination of *Tnfrsf11b* mRNA levels by real time qRT-PCR. Each value represents the mean±s.e.m. of three separate experiments. *P<0.05, significantly different from each control value. A representative result of three individual experiments is shown. (B) Effects of the overexpression of Bmal1 and CLOCK in MC3T3-E1 cells. MC3T3-E1 cells were transiently transfected with the expression vectors of Bmal1 and Clock, followed by further cultivation for 48 h and the subsequent determination of *Tnfrsf11b* levels by real time qRT-PCR. Relative mRNA expression was normalized to *Gapdh* expression. Each value represents the mean±s.e.m. of three separate experiments. *P<0.05, significantly different from each control value. (C) Per2-knockdown by siRNA in MC3T3-E1 cells. MC3T3-E1 cells were treated with *Per2* siRNA (siRNA-*Per2*) or non-silencing RNA (siRNA-*Negative*), followed by further cultivation for 48 h and the subsequent determination of *Tnfrsf11b* mRNA levels by real time qRT-PCR. Each value represents the mean±s.e.m. of three separate experiments. *P<0.05, significantly different from each control value. A representative result of three individual experiments is shown.

We investigated whether REV-ERBα regulated *Tnfrsf11b*, which encodes osteoprotegerin (OPG), in MC3T3-E1 osteoblastic cells. The treatment of MC3T3-E1 cells with GSK4112 significantly decreased the expression of *Tnfrsf11b* mRNA in a concentration-dependent manner (Fig. 2). Furthermore, significant increases were observed in the expression of *Tnfrsf11b* after 4 and 8 h in MC3T3-E1 osteoblastic cells treated with the synthetic REV-ERBα antagonist SR8278 (Fig. 3A). Conditioned media were collected from MC3T3-E1 cells treated with 10 µM SR8278 or DMSO, and OPG levels were then determined using ELISA. The results obtained showed that the secretion of OPG in MC3T3-E1 cells was significantly greater following the 24-h exposure to SR8278 than with the control treatment, which indicated that REV-ERBα negatively regulated the expression of *Tnfrsf11b* in MC3T3-E1 cells (Fig. 3B). We then attempted to elucidate the mechanisms regulating *Tnfrsf11b* gene expression in MC3T3-E1 cells transfected with small interfering RNA (siRNA) for the knockdown of *Bmal1* expression. Cells were transfected with siRNA for *Bmal1*, and *Tnfrsf11b* levels were determined by real time qRT-PCR. The results obtained demonstrated that *Tnfrsf11b* levels were significantly decreased in MC3T3-E1 cells 30 and 48 h after the transfection of *Bmal1* siRNA (Fig. 4A). Furthermore, the forced overexpression of the Bmal1–CLOCK complex (Bmal1–CLOCK) significantly increased the expression of *Tnfrsf11b* in MC3T3-E1 cells (Fig. 4B). The expression of *Tnfrsf11b* was also significantly increased by *Per2* siRNA (Fig. 4C), which indicated that the Bmal1–CLOCK heterodimer was involved in the regulation of *Tnfrsf11b* in osteoblastic cells. Taken together, these results suggested that the rhythmic expression of *Tnfrsf11b* in osteoblasts was regulated by the intrinsic circadian clock underlying the core loop by Bmal1–CLOCK transactivation of the nuclear receptor REV-ERBα, which provided feedback to repress the transcription of Bmal1.

### α_1B_-adrenergic receptor signaling was required for circadian regulation of *Tnfrsf11b* gene expression

**Fig. 5.**
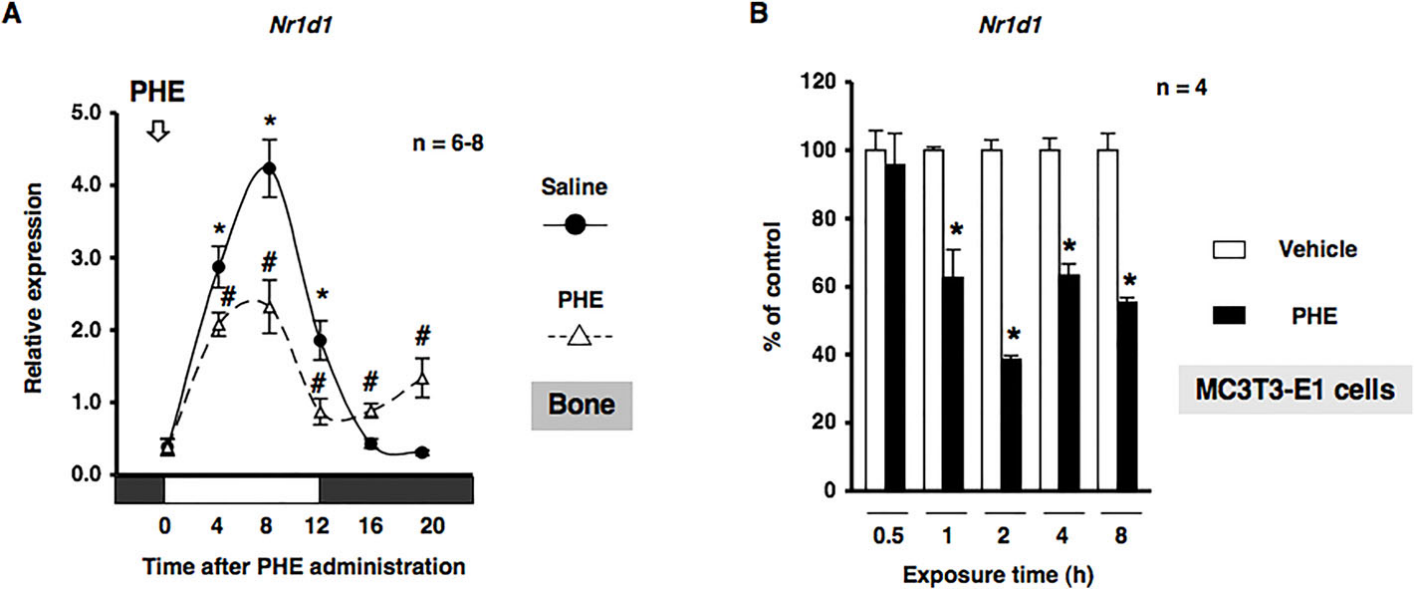
**α_1_-AR signaling regulated *Nr1d1* gene expression.** (A) The effects of the intraperitoneal administration of PHE at 10 µg/g on the rhythmic expression of *Nr1d1* mRNA in bone are shown. C57BL/6J mice were maintained under a 12:12-h light/dark cycle for 2 weeks and PHE were then administrated intraperitoneally at ZT0. Total RNA was isolated from the femurs (cancellous and cortical bone) of saline-treated and PHE-treated C57BL/6J mice. The mRNA levels of *Nr1d1* were determined by real time qRT-PCR using specific primers. Each value is the mean±s.e.m. (*n*=6 or 8 in each group). *P<0.05, significantly higher than the lowest value in a phase. #*P*<0.05, significantly different from each control value. The arrow indicates PHE administration. White boxes, light period; black boxes, dark period. (B) Cells were treated with 10 µM PHE for 0.5, 1, 2, 4, and 8 h, harvested, and then processed for real time qRT-PCR. Each value represents the mean±s.e.m. of four separate experiments. *P<0.05, significantly different from each control value obtained in MC3T3-E1 cells cultured in the absence of PHE.

**Fig. 6.**
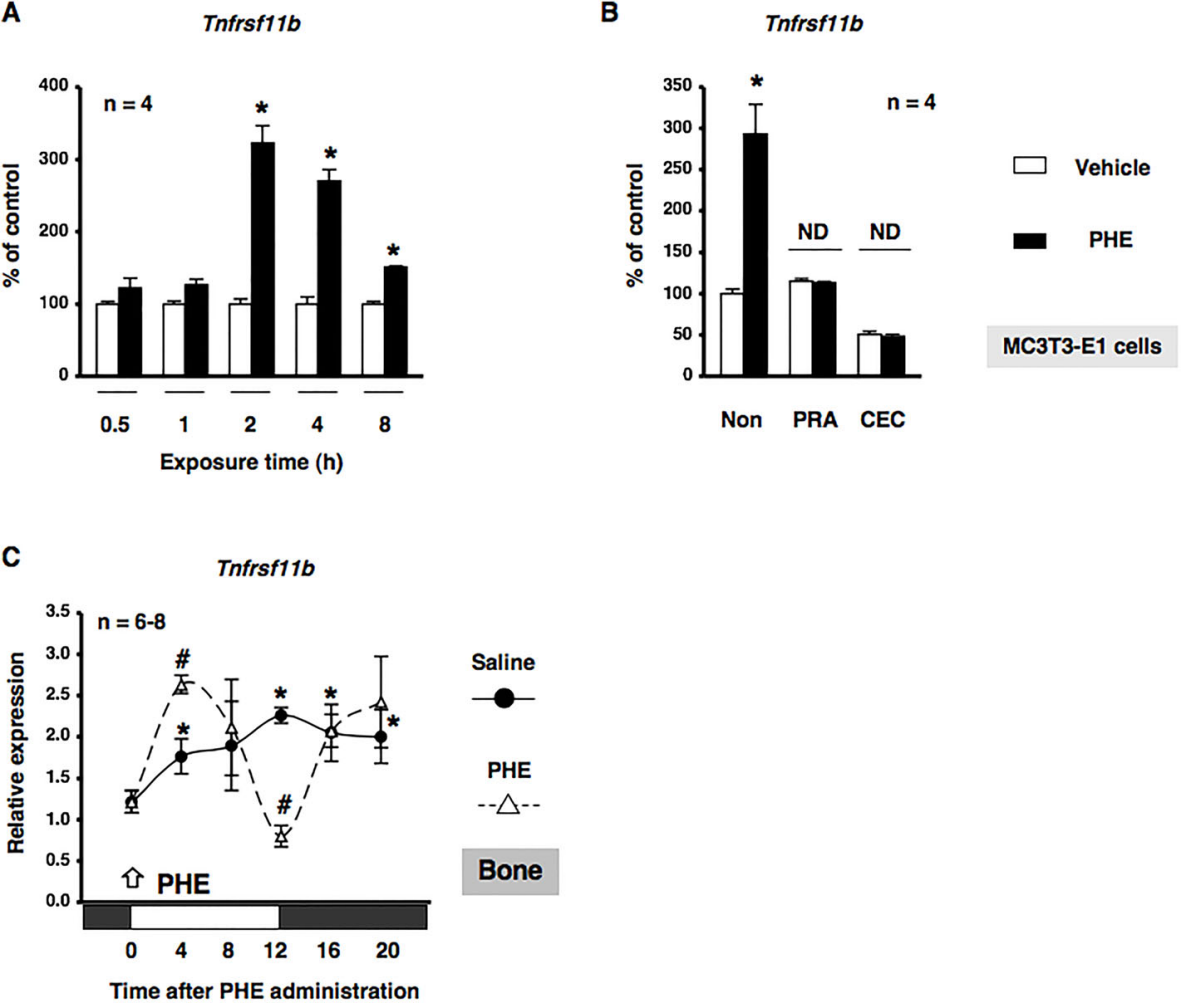
**α_1B_-AR signaling regulated *Tnfrsf11b* gene expression in osteoblasts.** (A) Cells were treated with 10 µM PHE for 0.5, 1, 2, 4, and 8 h, harvested, and then processed for real time qRT-PCR. Each value represents the mean±s.e.m. of four separate experiments. *P<0.05, significantly different from each control value obtained in MC3T3-E1 cells cultured in the absence of PHE. (B) Effects of the pretreatments with prazosin (PRA): a selective α_1_-AR antagonist, or chloroethylclonidine (CEC): an α_1B_-adrenoceptor-selective antagonist, on PHE-mediated *Tnfrsf11b* expression in MC3T3-El osteoblastic cells. Each inhibitor was administered 15 min before, cells were then treated with 10 µM PHE for 2 h, harvested, and processed for real time qRT-PCR. These transcription levels were achieved using specific primers for *Tnfrsf11b*. Relative mRNA expression was normalized to *Gapdh*. Each value represents the mean±s.e.m. of four separate experiments. *P<0.05, significantly different from the control value obtained in the absence of PHE. NS, not significant. (C) The effects of the intraperitoneal administration of PHE at 10 µg/g on the rhythmic expression of *Tnfrsf11b* mRNA in femurs (cancellous and cortical bone) are shown. C57BL/6J mice were maintained under a 12:12-h light/dark cycle for 2 weeks and PHE was then administrated intraperitoneally at ZT0. Total RNA was isolated from the distal region of the femurs of saline-treated and PHE-treated C57BL/6J mice. mRNA levels of *Tnfrsf11b* were determined by real time qRT-PCR using specific primers. Each value is the mean±s.e.m. (*n*=6 or 8 in each group). *P<0.05, significantly higher than the lowest value in a phase. #*P*<0.05, significantly different from each control value. The arrow indicates PHE administration. White boxes, light period; black boxes, dark period.

**Fig. 7.**
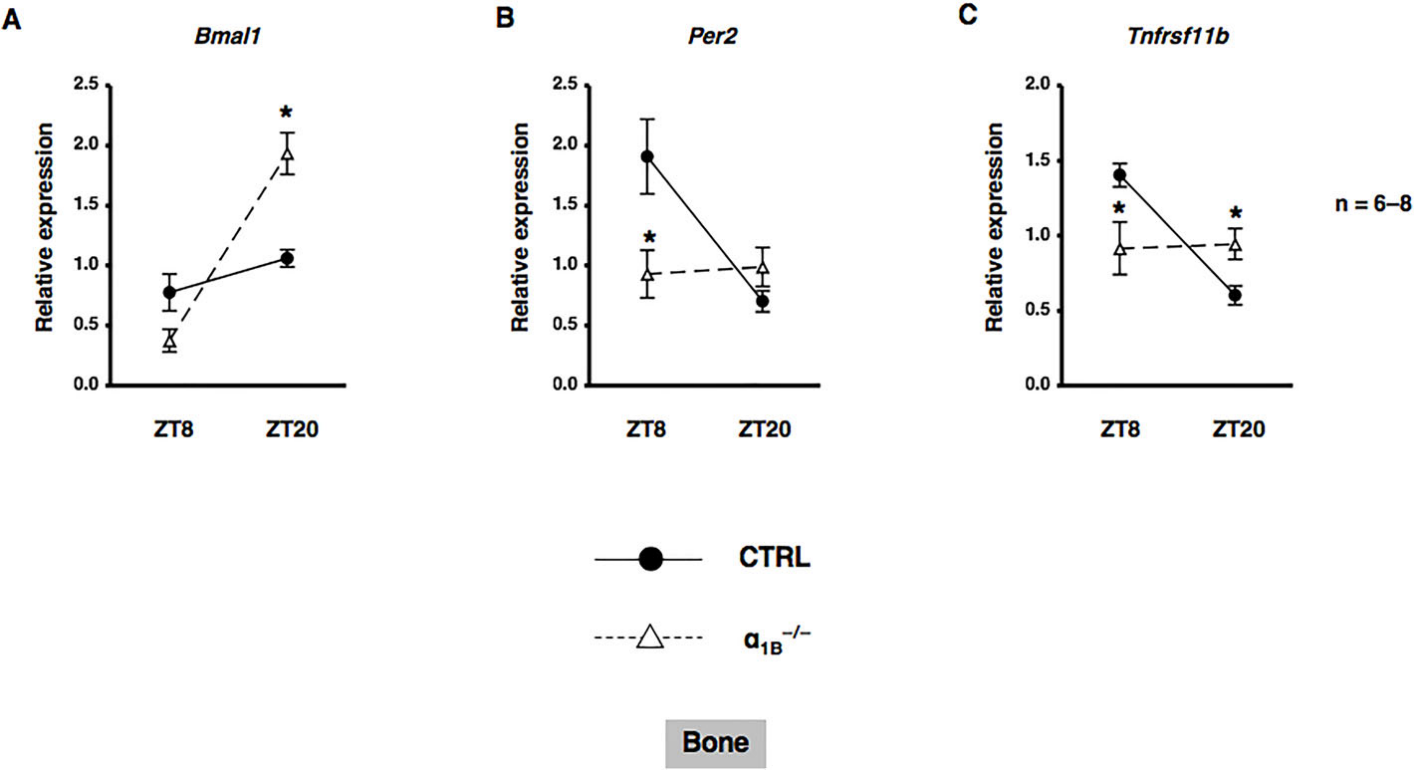
**α1B-AR signaling controlled circadian expression of the *Tnfrsf11b* gene in bone.** Total RNA was isolated from the distal ends of the femurs of α1B-AR-deficient and WT mice on ZT8 and ZT20, and the mRNA expression of *Bmal1* (A), *Per2* (B), and *Tnfrsf11b* (C) was then analyzed. Each value is the mean±s.e.m. (*n*=6 or 8 in each group). *P<0.05, significantly different from WT mice.

Based on previous findings in which sympathetic signaling through α_1_-AR entrained circadian oscillators in osteoblasts (Hirai et al., 2014a), we determined whether α_1_-AR signaling in osteoblasts mediated the rhythmic expression of *Nr1d1*. MC3T3-E1 cells were treated with PHE at 10 µM and mRNA expression was determined by real-time qRT-PCR analyses at the indicated time points between 12 h and 56 h. The exposure to PHE entrained *Nr1d1* with rhythmic expression in MC3T3-E1 osteoblastic cells, which indicated that α_1_-AR signaling reset the circadian clock in MC3T3-E1 osteoblasts (Fig. S1). We then evaluated the expression of *Nr1d1* in bone after the administration of PHE. We found that *Nr1d1* mRNA expression peaked near ZT20 in a 24-h rhythm in bone after the administration of saline (Fig. 5A). In addition, the rhythmic expression of *Nr1d1* in bone was altered by the systemic administration of 10 µg/g PHE at ZT0. The expression of *Nr1d1* mRNA was significantly decreased at ZT4, ZT8, and ZT12, and significantly increased at ZT16 and ZT20 after the systemic administration of PHE (Fig. 5A). We next characterized the expression of *Nr1d1* mRNA by α_1_-AR signaling in MC3T3-E1 osteoblastic cells. Total RNA was extracted from MC3T3-E1 osteoblastic cells following exposure to PHE and was subsequently analyzed by real time qRT-PCR. As shown in Fig. 5B, the expression of *Nr1d1* mRNA was significantly decreased by the exposure of MC3T3-E1 osteoblastic cells to PHE for 1, 2, 4, and 8 h, which indicated that α_1_-AR signaling negatively regulated the expression of *Nr1d1* in osteoblasts. Our results indicated that α_1_-AR signaling in osteoblasts mediated rhythmic *Tnfrsf11b* expression in osteoblasts; therefore, we characterized the expression of *Tnfrsf11b* mRNA in response to the PHE stimulation in MC3T3-E1 osteoblastic cells. The results obtained showed that the expression of *Tnfrsf11b* mRNA was significantly increased by the exposure of MC3T3-E1 osteoblastic cells to PHE for 2, 4, and 8 h (Fig. 6A). In addition, the pretreatment with the α_1_-AR antagonist prazosin (PRA) or α_1B_-adrenoceptor-selective antagonist chloroethylclonidine (CEC) completely inhibited PHE-induced *Tnfrsf11b* expression, as determined by a real time qRT-PCR analysis of MC3T3-E1 osteoblastic cells, which suggested that PHE-induced *Tnfrsf11b* expression in osteoblasts was mediated by α_1B_-AR signaling (Fig. 6B). In order to obtain a deeper understanding of the physiological function of α_1B_-AR signaling on the circadian expression of *Tnfrsf11b*, we evaluated the expression of *Tnfrsf11b* in bone after the administration of PHE. Consistent with previous findings (Fujihara et al., 2014), the rhythmic expression of *Tnfrsf11b* peaked near ZT12 in a 24-h rhythm in bone after the administration of saline (Fig. 6C). The administration of 10 µg/g PHE at ZT0 significantly increased the expression of *Tnfrsf11b* at ZT4 in bone. In contrast, the expression of *Tnfrsf11b* was decreased significantly at ZT12 after the systemic administration of PHE (Fig. 6C), which indicated that the *Tnfrsf11b* gene exhibited a rhythmic expression pattern over 24 h and was regulated in part by α_1B_-AR signaling in osteoblasts. Furthermore, to determine the importance of α_1B_-AR signaling in regulating the rhythmic expression of *Tnfrsf11b*, we attempted to elucidate the effects of the knockout of α_1B_-AR on *Bmal1*, *Per2*, and *Tnfrsf11b* mRNA expression in bone. We previously demonstrated that the expression of *Bmal1* and *Per2* displayed a circadian pattern in cancellous and cortical bone (Hirai et al., 2014b). The expression of *Bmal1* mRNA was significantly lower at ZT8, but was significantly higher at ZT20 in α_1B_-AR-deficient mice (α_1B_^−/−^) than in wild-type (WT) mice (Fig. 7A). Additionally, the rhythmicity of *Per2* was abolished in the bone of α_1B_^−/−^ mice (WT: ZT8, 1.91±0.31; ZT20, 0.70±0.09; *P*<0.05. α_1B_^−/−^: ZT8, 0.93±0.20; ZT20, 0.99±0.16; no significant difference), indicating that the ablation of α_1B_-AR signaling altered the molecular clock in bone-related cells (Fig. 7B). Furthermore, the results obtained showed that the expression of *Tnfrsf11b* in the bone of WT was significantly lower at ZT20 than at ZT8 (Fig. 7C), whereas no significant differences were observed in *Tnfrsf11b* expression levels between ZT8 and ZT20 in α_1B_^−/−^ mice (Fig. 7C). Taken together, these results suggested that the sympathetic nervous system through α_1B_-AR controlled the rhythmic expression of *Tnfrsf11b* by regulating the molecular clock in osteoblasts.

## DISCUSSION

We herein identified *Tnfrsf11b* as one of the CCGs in osteoblasts. The cellular rhythmic expression of *Tnfrsf11b* in osteoblasts was found to be controlled by the molecular clock, especially its core loop component Bmal1 and REV-ERBα. Moreover, the sympathetic signal through α_1B_-AR regulated the expression of *Tnfrsf11b* in osteoblasts. To the best of our knowledge, this is the first study to demonstrate that α_1B_-AR signaling systematically drove the rhythmic expression of *Tnfrsf11b* by regulating clock genes, thereby showing the physiological significance of the circadian clockwork in bone remodeling.

**Fig. 8.**
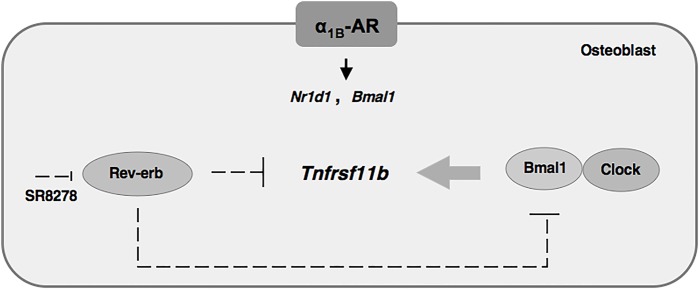
**Regulation of *Tnfrsf11b* expression by the circadian core clock in osteoblasts.** The rhythmic *Tnfrsf11b* expression was mediated by an interplay between the Rev-erbα and Bmal1 clock genes. In addition, α1B-AR signaling in osteoblasts regulates the circadian expression of *Tnfrsf11b* by regulating the expression of *Nr1d1* and *Bmal1*.

The potential of OPG to demonstrate a possible circadian rhythm was reported previously in humans (Joseph et al., 2007). Several recent studies showed that circadian clock genes were rhythmically expressed in the bone of mice (Fujihara et al., 2014; Hirai et al., 2014b). Additionally, the molecular mechanisms underlying cell-autonomous circadian clocks were found to be composed of transcriptional-translational feedback loops involving clock genes through E-box elements located in their promoter regions in osteoblasts (Hirai et al., 2014b) as well as other cell types (Sato et al., 2006). In the present study, we identified, for the first time, *Tnfrsf11b* as a CCG regulated by circadian oscillators in osteoblasts. On a molecular level, loss-of-function and gain-of-function experiments both showed that the rhythmic expression of *Tnfrsf11b* was generated by its own transcriptional/translational feedback loop. This feedback loop may involve a set of clock genes that are regulated by the Bmal1–CLOCK heterodimer in MC3T3-E1 osteoblastic cells. The results of the present study also revealed that the rhythmic expression of *Nr1d1* peaked near ZT8 in a 24-h rhythm in bone. REV-ERBα, which is a circadian transcriptional repressor, has been shown to negatively regulate *Bmal1* transcription by competing for shared RORE promoter elements and has a key role in several metabolic pathways (Cho et al., 2012; Marciano et al., 2014). The results of the pharmacological experiments showed that the REV-ERB agonist GSK4112 suppressed the expression of *Tnfrsf11b* as well as *Bmal1*, whereas the down-regulation of REV-ERBα by SR8278 induced *Tnfrsf11b* and the production of OPG in MC3T3-E1 osteoblastic cells (Figs 1 and 3), suggesting that REV-ERBα negatively regulated the expression of *Tnfrsf11b* in osteoblasts. Our results indicated that REV-ERBα has emerged as a critical component of the core circadian feedback loop controlling the cyclic expression of *Bmal1* in osteoblasts. Furthermore, the rhythmic expression of *Tnfrsf11b* in osteoblasts was driven by clock genes such as REV-ERBα and Bmal1 (Fig. 8). Although Bmal1 is involved in regulation of the expression of *Tnfrsf11b*, the regulatory mechanisms of *Tnfrsf11b* are not completely understood so far. One possible examination is that the Bmal1–CLOCK complex directly binds to DNA enhancers of *Tnfrsf11b* gene and drives circadian oscillation of transcription. Another possibility is that the Bmal1–CLOCK complex acts indirectly through the transactivation of other transcription factors. Recent studies reported that indirect regulation through transcriptional and posttranscriptional events plays a key role in generating diverse phases of gene expression (Yoshitane et al., 2014). Therefore, it would suggest that indirect transcriptional and posttranscriptional regulations play key roles in the regulation of rhythmic expression of *Tnfrsf11b* in osteoblasts.

Recent studies reported that β-AR signaling regulated the circadian expression of the clock genes *PER1*, *Per2*, *PER3*, and *Bmal1* in human osteoblasts (Komoto et al., 2012). AR signaling regulates the *PTGS2* gene encoding prostaglandin G/H synthetase 2, which is a late-limiting enzyme for the synthesis of prostaglandins, by driving clock genes in osteoblasts (Hirai et al., 2014b). On the other hand, the physiological action of epinephrine was previously found to be mediated by α_1_-AR signaling as well as β-AR signaling in osteoblasts (Takeuchi et al., 2001; Togari and Arai, 2008). We also reported the expression of *BMP4* in osteoblasts (Hirai et al., 2014a). In the present study, we showed that PHE stimulated oscillations in the *Nr1d1* gene in MC3T3-E1 osteoblastic cells, and altered its rhythmicity in bone (Fig. S1 and Fig. 5A), suggesting that α_1_-AR signaling is a potential signal that alters the expression of the molecular clock and CCGs in osteoblasts. Consistent with our previous *in vitro* findings (Takeuchi et al., 2001), the results of the pharmacological experiments indicated that *Tnfrsf11b* was up-regulated by α_1B_-AR signaling in MC3T3-E1 osteoblastic cells (Fig. 6A,B). Additionally, the administration of PHE stimulated the expression of *Tnfrsf11b in vivo* (Fig. 6C). Therefore, these results indicated that the *Tnfrsf11b* in bone is synchronized by the sympathetic nervous system via α_1B_-AR. We herein employed α_1B_^−/−^ mice to address the importance of endogenous regulators of the molecular clock in regulating *Tnfrsf11b* expression in cancellous and cortical bone. The results of this study showed that the ablation of α_1B_-AR signaling altered the expression of *Tnfrsf11b*, *Bmal1*, and *Per2* in cancellous and cortical bone (Fig. 7). Previous studies, including ours, reported that the mRNAs of α_1B_-AR subtypes were expressed in human osteoblasts (Togari et al., 1997; Huang et al., 2009), but not in osteoclasts (data not shown). Thus, regulation of the molecular circadian clock by α_1B_-AR signaling was required for the circadian regulation of *Tnfrsf11b* in osteoblasts (Fig. 8). In the present study, we also showed that Bmal1–CLOCK complex regulated the expression of *Tnfrsf11b* in MC3T3-E1 osteoblastic cells. Therefore, β-AR signaling in osteoblast may also regulate the circadian expression of *Tnfrsf11b* by clock genes including Bmal1. The exact mechanism underlying the interaction of β-AR signaling in osteoblasts with clock genes including Bmal1 on *Tnfrsf11b* expression, however, remains to be elucidated in future studies.

The circadian clock was recently shown to control many aspects of energy metabolism, the immune system, and cardiovascular physiology (Maury et al., 2014; Kashiwada et al., 2011; Takeda and Maemura, 2010). These studies demonstrated that the genetic and pharmacological perturbation of clock genes led to obesity, diabetes, immune dysfunctions, and hypertension, suggesting that dysregulation of the circadian molecular clock contributes to the pathogenesis of these diseases (Solt et al., 2012; Yu et al., 2013; Anea et al., 2009). Osteoclast resorptive activity also exhibits circadian rhythmicity and is controlled by various endocrine hormones and cytokines (Jubiz et al., 1972; el-Hajj Fuleihan et al., 1997; Joseph et al., 2007). This study demonstrated the rhythmic expression of *Tnfrsf11b* mRNA levels in osteoblasts, indicating that circadian oscillators in osteoblasts modulated diverse physiological processes in bone remodeling and disruption of the clockwork may contribute to the pathogenesis of bone diseases such as osteoporosis. Several lines of evidence have demonstrated that the RANKL–RANK–OPG system plays critical roles in bone homeostasis. Previous report showed that α_1_-AR stimulated the *Tnfsf11*, which encodes RANKL, in MC3T3-E1 osteoblastic cells (Nishiura and Abe, 2007; Huang et al., 2009). In addition, we have showed the rhythmic expression of *Tnfsf11* mRNA levels as well as *Tnfrsf11b* in bone (Fujihara et al., 2014). Therefore, α_1_-AR signaling might play a role in the regulation of rhythmic expression of *Tnfsf11* in bone-related cells. Further studies are necessary to investigate these processes.

Osteoblasts synthesize and regulate the deposition and mineralization of the extracellular matrix. MC3T3-E1 cells, derived from newborn murine calvariae and as used in the current study, closely resemble osteoblasts. They express osteoblast-characteristic genes and are able to undergo osteoblastic differentiation under appropriate conditions (Sudo et al., 1983; Quarles et al., 1992). Although this *in vitro* cell culture system is a suitable model for studying the clockwork in osteoblasts, further studies using other osteoblast culture systems such as primary cultured osteoblasts are needed to confirm these findings.

In conclusion, we demonstrated that the circadian core loop component Bmal1 and REV-ERBα were involved in the regulation of *Tnfrsf11b* gene expression in MC3T3-E1 osteoblastic cells. In addition, our results suggest that α_1B_-AR signaling drives the circadian rhythmicity of *Tnfrsf11b* to regulate the expression of REV-ERBα and Bmal1 in osteoblasts. These results may contribute to a deeper understanding of the mechanisms underlying the molecular clock in bone remodeling.

## MATERIALS AND METHODS

### Mice

C57BL/6J mice were originally obtained from Japan SLC, Inc. (Hamamatsu, Japan). α_1B_-AR-deficient mice (α_1B_^−/−^) were provided by CARD (Center for Animal Resources and Development, Kumamoto University, Japan). The generation of α_1B_^−/−^ was described previously (Cavalli et al., 1997). α_1B_^−/−^ had been backcrossed onto the C57BL/6J background for more than five generations. We used α_1B_^−/−^ and their wild-type (WT) littermates. The genotypes of the offspring were screened using PCR. All mice were treated in accordance with the Guidelines for Animal Experiments at the School of Dentistry, Aichi-Gakuin University. Food and water were available *ad libitum*. Animals were housed together in automatically controlled conditions of temperature (23±1°C) and humidity (50±10%) under a 12-h light:dark cycle (Hirai et al., 2014a,b).

### Drugs and treatment

Prazosin, a selective α_1_-AR antagonist, chloroethylclonidine (CEC), an α_1B_-adrenoceptor-selective antagonist, GSK4112, a REV-ERB agonist, and SR8278, a REV-ERB antagonist, were purchased from Sigma-Aldrich (St. Louis, MO, USA). α_1_-AR pathways were stimulated using phenylephrine (PHE), a nonspecific α_1_-AR agonist (Sigma-Aldrich). Eight-week-old male C57BL/6J mice were randomized by weight, assigned to groups, and acclimated to their cages for 2 weeks prior to the experiment. Bone tissue samples were dissected and kept at −80°C for total RNA until assayed (Hirai et al., 2014b).

### Cell cultures and transfection

MC3T3-E1 cells were purchased from the RIKEN Cell Bank. MC3T3-E1 cells were cultured in α-MEM containing 10% FBS and 1% penicillin/streptomycin at 37°C in a 5% CO_2_ atmosphere. To induce differentiation, the culture medium was replaced with α-MEM containing 50 µg/ml ascorbic acid and 5 mM β-glycerophosphate. The culture medium was changed every 2-3 days. The plasmids used in this study were obtained from Addgene. mCLOCK and mBmal1 cDNA were cloned into pcDNA4 to produce pCKPC4, which expresses hexahistidine- and Flag-tagged mCLOCK (pcDNA-CLOCK), and also into pcDNA3 to produce pBMPC3, which expresses hexahistidine-tagged mBmal1 (pcDNA-Bmal1). MC3T3-E1 cells were plated at a density of 1.0×10^5^ cells/cm^2^. Cells were transfected after 24 h with pcDNA-Bmal1 and pcDNA-CLOCK, or the empty vector using FugeneHD reagent (Promega, Madison, WI, USA) according to the manufacturer's instructions.

### siRNA nucleofection

MC3T3-E1 cells were grown in α-MEM supplemented with 10% FBS and 1% penicillin/streptomycin to ∼70% confluency, followed by transient transfection with either siRNA targeting Bmal1 or non-silencing RNA diluted in Opti-MEM using Lipofectamine RNAiMAX (Invitrogen Life Technologies, Carlsbad, CA, USA) according to the manufacturer's protocol. Silencer Select siRNAs were used (Applied Biosystems, Thermo Fisher Scientific by Life Technologies, Carlsbad, CA, USA). Bmal1 siRNAs and non-silencing RNA were both used at final concentrations of 10 nM. The medium was then replaced with fresh medium. Cells were harvested for total RNA extraction at the indicated time points.

### RNA extraction and real time PCR

As *in vivo* experiments, total RNA was extracted from femur (cancellous and cortical bone). As *in vitro* experiments, total RNA samples were extracted from the cells. Total RNA was isolated with an RNeasy Mini Kit (Qiagen, Valencia, CA, USA) according to the protocol of the manufacturer. One microgram of RNA was reverse transcribed into cDNA using the QuantiTect Reverse Transcription Kit according to the protocol of the manufacturer (Qiagen). Gene expression was analyzed with the Step-One-Plus real-time PCR system with Step One Software v2.0 (Applied Biosystems). Reactions were performed in 20-µl volumes using a QuantiTect SYBR Green PCR Kit (Qiagen). Cycling conditions were 50°C for 2 min, 95°C for 10 min, followed by 40 cycles of 95°C for 15 s and 60°C for 1 min. The relative quantity for each sample was normalized to the average level of the constitutively expressed housekeeping gene *Gapdh*. The following primers were used: *Gapdh*, forward 5′-TGGAGAAACCTGCCAAGTATG-3′, reverse 5′-GGAGACAACCTGGTCCTCAG-3′; *Tnfrsf11b*, forward 5′-CACTCGAACCTCACCACAGAG-3′, reverse 5′-TCAATCTCTTCTGGGCTGATCTTC-3′; *Nr1d1*, forward 5′-TGGCCTCAGGCTTCCACTATG-3′, reverse 5′-CCGTTGCTTCTCTCTCTTGGG -3′; *Bmal1*, forward 5′-GCCGAATGATTGCTGAGGAA-3′, reverse 5′-GGGAGGCGTACTCGTGATGT-3′; *Per2*, forward 5′-ATGCTCGCCATCCACAAGA, reverse 5′-GCGGAATCGAATGGGAGAAT-3′.

### OPG ELISA

Mouse OPG in conditioned media was assayed using the Quantikine M Elisa kit (R&D Systems, Minneapolis, MN, USA). The medium was collected from cultures of MC3T3-E1 cells and subjected to ELISA. Optical density was read at 450 nm with a correction wavelength of 540 nm.

### Data analysis

All data are expressed as the mean±s.e. Two-tailed Student's *t*-test combined with Bonferroni's correction following a one-way analysis of variance was used for multiple comparisons. Differences with *P* values <0.05 were considered significant.
